# Functionality, *in Vitro* Digestibility and Physicochemical Properties of Two Varieties of Defatted Foxtail Millet Protein Concentrates

**DOI:** 10.3390/ijms10125224

**Published:** 2009-12-01

**Authors:** Tabita Kamara Mohamed, Kexue Zhu, Amadou Issoufou, Tarawalie Fatmata, Huiming Zhou

**Affiliations:** 1 State Key Laboratory of Food Science and Technology, Jiangnan University, No 1800, Lihu Road, Wuxi, 214122 Jiangsu, China; 2 Milton Margai College of Education and Technology, Njala University, Goderich Campus, Freetown, Sierra Leone

**Keywords:** *in vitro* trypsin digestibility, functionality, differential scanning calorimetry, millet protein concentrates

## Abstract

Two varieties of foxtail millet protein concentrates (white and yellow) were characterized for *in vitro* trypsin digestibility, functional and physicochemical properties. Millet protein concentrate was easily digested by trypsin *in vitro*. Essential amino acids were above the amounts recommended by the Food Agricultural Organization/World Health Organization (FAO/WHO/UNU) for humans. Yellow millet protein concentrate (YMPC) possessed the highest differential scanning calorimetry result (peak temperature of 88.98 °C, delta H = 0.01 J/g), white millet protein concentrate (WMPC) had the lowest (peak temperature 84.06 °C, delta H = 0.10 J/g). The millet protein concentrates had molecular sizes around 14.4 and 97.4 kDa. They have U-shape solubility curves. Water-binding capacity was in the range of 5.0 and 7.0 g/g, while oil absorption capacity ranged between 4.8 and 5.9 g/g. WMPC had higher bulk density (0.22 g/mL) and emulsifying capacity than YMPC and Soy Protein Concentrate (SPC). Foam capacity and foam stability ranged from 137 to 73 g/mL for WMPC, from 124 to 61 g/mL SPC and from 124 to 46 g/mL for YMPC. Millet protein concentrates are potential functional food ingredients.

## 1. Introduction

Plant proteins are an abundant and relatively inexpensive source of proteins that are widely recognized for their high nutritional value and excellent functional properties. The functional properties of plant proteins have been exploited in a multitude of applications (for example, solubility in beverages, foaming in whipped toppings, and emulsification in processed meat) and had resulted into an ever increasing demand for plant protein ingredients with improved processing and functional characteristics. This potential usefulness, however, will also depend on their functional properties, which affect the sensory characteristics of food and play an important role in the physical behavior of food or its ingredients during preparation, processing, and storage. Functional properties include emulsification, foam formation, viscosity, improvement of appearance, texture, and water holding and oil holding capacities. On the basis of these properties, the specific protein selected to be used in a certain food will depend on its required function in the final product [1,2]. Functional properties are, however, often used to denote any property of proteins that affects their use, either as a processing aid or as a direct contributor of product attributes [3].

Millets typically contain higher quantities of essential amino acids and are higher in fat content than maize, rice, and sorghum [4]. Millet contains 12.3% crude protein and 3.3% minerals. [5]. The main components of millet include starch, protein, lipid, vitamins and minerals [6]. It was further reported that minerals like, magnesium, manganese and phosphorus were significantly higher than the others [7]. Foxtail millet (*Setaria italica* L.) is also known as Italian millet and is one of the world’s oldest cultivated crops. In the northern area of China it has been widely used as a nourishing gruel or soup for pregnant and nursing women, and has been applied in food therapy [8]. Foxtail millet is also an important cereal and nutritious food in traditional diets, especially for people in Europe, Asia and Africa continents.

The digestibility of the nutrients must be known in order to evaluate fully the significance of nutrient concentration. Protein functional properties are determined to a large extent by a protein’s physicochemical and structural properties [9]. Protein solubility is an important prerequisite for food protein functional properties, and it is a good index of potential applications of proteins [10]. Researchers have reported that protein solubility has a close relationship with emulsifying properties [11] and foaming properties [11,12]. Bulk density is an important parameter that determines the packaging requirement of a product [13]. Proteins isolates are the basic functional components of various high protein processed food products and thus determine the textural and nutritional properties of the foods. [11,14]. Therefore, the objective of this study was to investigate the *in vitro* digestibility, amino acid composition, differential scanning calorimetry (DSC), protein solubility, molecular size, foaming, emulsifying, bulk density water and oil holding capacity of the two varieties of defatted foxtail millet protein concentrates compared to commercial soy protein concentrate.

## 2. Experimental Section

### 2.1. Materials and Methods

The two varieties of foxtail millet (white and yellow) were purchased from a local market in Wuxi, China. All chemicals used in the experiments were of analytical grade. The foxtail millet (1 kg) was defatted twice with hexane for 8 hours at room temperature. The defatted white millet flour (DWMF) and the defatted yellow millet flour (DYMF) were air-dried for 24 hours under a vacuum drier. The defatted flour was milled using a laboratory-scale hammer miller and the resulting flour was sieved through a 60 mesh screen and stored at 5 °C in sealed glass jars until used. The commercial soy protein concentrate was purchased from a supermarket in China, produced by Fuxin Flour Mill Co. Ltd (Shanghai, China).

### 2.2. Proximate Analysis

The proximate composition of white millet flour (WMF), yellow millet flour (YMF), DWMF and DYMF was determined according to the method described by Ceirwyn [15]. The moisture content was determined by drying in an oven at 105 °C until a constant weight was obtained. Ash was determined by weighing the incinerated residue obtained at 525 °C after 4 hours. Crude fat was extracted by the Soxhlet method with petroleum ether. The crude protein was determined by the micro-Kjeldahl method and a Conversion factor of 6.25 was used to quantify the crude protein content [15]. The carbohydrate content was estimated by subtracting the sum of percentage of moisture, crude fat, crude protein, ash and crude fibre contents from 100%.

### 2.3. Crude Fibre Determination

The sample (2 g) was accurately weighed into the fibre flask and 100 mL of 0.25 N H_2_SO_4_ was added. The mixture was heated under reflux for 1 hour with the heating mantle. The hot mixture was filtered through a fibre sieve cloth. The filterate obtained was discarded and the residue was returned to the fibre flask to which 100 mL of (0.31 N NaOH) was added and heated under reflex for another 1 hour. The mixture was filtered through a fibre sieve cloth and 10 mL of acetone added to dissolve any organic constituents. The residue was washed twice with about 50 mL hot water on the sieve cloth before it was finally transferred into a crucible. The crucible and the residue were oven-dried at 105 °C overnight to drive off moisture. The oven dried crucible containing the residue was cooled in a dessicator and later weighed to obtain the weight W_1_. The crucible with weight W_1_ was transferred to the muffle furnace for ashing at 550 °C for 4 hours. The crucible containing white or grey ash (free of carbonaceous material) was cooled in the dessicator and weighed to obtain W_2_. The difference W_1_–W_2_ gives the weight of fibre (AOAC, 1990). The percentage fibre was obtained by the formula:

(1) $$\%\;\text{Fibre}={\text{W}}_{1}-{\text{W}}_{2}\times 100$$

### 2.4. Preparation of Protein Concentrates

Defatted millet flour protein concentrate was prepared according to the procedure described by Olayide [16], with some modifications. The defatted flour was dispersed in distilled water at flour to water ratio of 1:5 (w/v); the pH was adjusted to pH 9.5 with 1 M NaOH and stirred for 3 hours at 30 °C. The extract was separated by centrifugation at 4,000 × g for 30 min at room temperature. The residues were re-extracted twice as described above. The extracts were then combined and protein was precipitated by adjusting the pH to 4.0 with 1 M HCl before centrifugation at 4,000 × g for 20 min. The protein concentrate (pH 4.2) was washed twice with distilled water. It was then resuspended in distilled water and the pH was adjusted to 7.0 with 1 M NaOH prior to freeze-drying. The dry protein concentrates were stored in airtight glass containers for subsequent analyses. The protein content was determined by the Kjeldahl method.

### 2.5. Amino Acid Analysis

For the determination of the amino acids, samples of protein concentrate (100 mg) for all the samples and 5 mL 6 M HCl were added to a 50 mL stopper bottle and sealed. The air was removed by keeping the sample in a vacuum chamber. The sealed samples were placed in an oven at 120 °C for 16 hours to hydrolyze. After hydrolysis, 5 mL of 2 mM norleucine internal standard was added and the solution was filtered in a 0.2 μL Gelman membrane filter. 1 mL of stock sample was pipetted into a 50 mL borosilicate glass serum bottle and dried in a freeze-drier. 1 mL of sodium diluent buffer (pH 2.2) was added to the freeze-dried residue and transferred to a 1.5 mL micro-centrifuge tube for HPLC analysis. The prepared samples were injected as 2.5 μL volumes and run on a Waters HPLC (Waters Corporation, Milford, MA, USA) at a flow rate of 0.4 mL/min with a Pickering sodium ion-exchange column of 4 × 150 mm (Pickering Laboratories, Inc., Mountain View, CA, USA) and sodium eluent (pH 3.15 and 7.40). TRIONE^®^ ninhydrin reagent was added with post column instrument (TRIONE^®^ ninhydrin derivatization instrument, Pickering Laboratories, Inc.). The light absorbance of amino acids was detected with an UV Visible detector (Pickering Laboratories Inc.) at 570 nm wavelength and the amino acids were quantified by comparing with standard amino acid profiles. Methionine and cysteine were determined separately by oxidation products according to the performic acid procedure of Moore [17] before hydrolysis in 6 M HCl. Tryptophan was determined after alkaline hydrolysis by isocratic ion-exchange chromatography with *o*-phthalaldehyde derivatization followed by fluorescence detection by Ravindran and Bryden [18]. Amino acid composition was reported as g/100 g of protein.

### 2.6. Sodium Dodecyl Sulfate Polyacrylamide Gel Electrophoresis (SDS-PAGE)

SDS-PAGE was done according to the method described by Laemmli [19], with 12% separating and 4% stacking gels using low molecular weight (14.4–97.4 kDa) markers obtained from Sigma Aldrich (St. Louis, MO, USA). For lyophilized crude extract powder; the (0.005 g) was dissolved in 1 mL of 20 mM Tris-HCl buffer at pH 6.8. The solution was then centrifuged at 12000 × g for two min in a Thermo Scientific Sorvall Legend Micro 17 centrifuges (Thermo Fisher Scientific Inc, Germany), to obtain the analytical sample. Sample buffer composition was prepared with 20 mL glycerol, 40 mL 10% (4.0%) 25 mL 0.5 m Tris; pH 6.8, and 15 mL distilled water. Running condition was 15 A for 30 min and increased to 30 A for the rest of the time, Time of migration was between 90 to 105 min and size of the gel is min gel. The gels were fixed and stained with 0.1% Coomassie Brilliant Blue R-250 in 10% acetic acid and 40% ethanol, then destained with 75 mL acetic acid (7.5%), 50 mL methanol (5%) and 875 mL distilled water (87.5%).

### 2.7. Protein Digestibility by Trypsin

*In vitro* digestibility was carried out according to the method described by Elkhalil *et al.* [20], with slight modifications. Twenty mg of protein concentrate samples were digested in triplicate in 10 mL of trypsin (0.2 mg/mL in 100 mM Tris–HCl buffer, pH 7.6). The suspension was incubated at 37 °C for 2 hours. Hydrolysis was stopped by adding 5 mL 50% trichloroacetic acid (TCA). The mixture was allowed to stand for 30 min at 4 °C and was then centrifuged at 9,500 × g for 30 min using a D-3756 Osterode AM Harz Model 4515 Centrifuge (Sigma, Germany). The resultant precipitate was dissolved in 5 mL of NaOH and protein concentrate was measured using the Kjeldahl method. Digestibility was calculated as follows:

(2) $$\text{Protein}\;\text{digestibility}\;(\%)=\frac{\left(A-B\right)}{A}\times 100$$

Where: A: total protein content (mg) in the sample.

B: total protein content (mg) in TCA precipitate.

### 2.8. Protein Solubility

Protein solubility was determined according to the procedure of Bera and Mukherjee [21], with slight modifications. One hundred mg of millet protein concentrates and soy protein concentrate were dispersed in 10 mL of distilled deionized water. The suspensions were adjusted to pH 2.0 up to pH 12.0 using either 0.1 M HCl or 0.1 M NaOH. These suspensions were shaken (Lab-Line Environ-Shaker; Lab-Line Instrument, Inc., Melrose Park, IL, USA) for 30 min at room temperature (approximately 25°C) and centrifuged at 4000 × g for 30 min. The protein content of the supernatant was determined by the Kjeldahl method and percent protein solubility was calculated as follows:

(3) $$\text{Protein}\;\text{solubility},\;\%=\frac{PS(mg)}{PIS(g)}\times 100$$

Where: PS: Amount of protein in supernatant.

PIS: Protein in initial sample.

### 2.9. Foaming Capacity (FC) and Foam Stability (FS)

Foaming capacity was evaluated by the method of Bernardi Don *et al.* [22], with minor modifications. Thirty mL of 30 g/L aqueous dispersion was mixed thoroughly using an Ultra-turrax T25 homogenizer at 9500 rpm for 3 min in a 250 mL graduated cylinder and the total volume of the liquid was measured immediately after 30 s. The difference in volume was expressed as the volume of the foam. Foam stability was estimated by measuring the fall in volume of the foam after 60 min.

### 2.10. Emulsifying Capacity

The emulsifying capacity was determined by the method of Yasumatsu *et al.* [23]. The sample (1.25 g) was homogenized with 50 mL of water for 30 s, with the use of a polyton homogenizer at 10,000 rpm/min. Pure soybean oil (25 mL) was added to each sample, and the mixtures were divided evenly into four tubes, and centrifuge at 1,100 × g for 5 min. emulsifying capacity was calculated by dividing the volume of the emulsified layer by the volume of emulsion before centrifugation and expressing the result as percentage.

### 2.11. Water/Oil Absorption Capacity

Sample (0.5 g) was taken and mixed with 3 mL of distilled water or refined groundnut oil. The slurry was centrifuged at 750 × g for 15 min. The pellet was drained for 30 min and the gain in weight per unit weight was reported as water or oil absorption capacity (g/g), respectively.

### 2.12. Bulk Density

A known weight of the protein concentrate was added to a graduated measuring cylinder. The cylinder was gently tapped and volume occupied by the sample was determined. Bulk density was reported as weight per unit volume (g/mL).

### 2.13. Differential Scanning Calorimetry (DSC)

Thermal properties of protein concentrates were evaluated using differential scanning calorimetry (Pyris-I-DSC, Perkin-Elmer Corp., Norwalk, CT, and USA). 70 mg of the various samples were dissolved in 1 mL of 0.05 M phosphate buffer (pH 7.0) containing 0.1 M NaCl. The protein solutions (45 μL) were transferred and hermetically sealed in a stainless steel pan. The samples were heated by scanning from 25 to 135 °C at a rate of 10 °C per min against a reference containing 45 μL buffers without protein in a differential scanning calorimeter (Perkin-Elmer Corp.) The denaturation peak temperature and enthalpy were calculated by a thermal analysis data software program.

### 2.14. Statistical Analysis

Data were analyzed by analysis of variance (ANOVA) using SAS statistical software package (v. 8.1, SAS Institute, Cary, NC, USA). Each value was determined by at least three replicates. Results were given as mean ± standard deviation.

## 3. Results and Discussion

### 3.1. Proximate Chemical Composition

The results of the proximate chemical composition of WMF, YMF, DWMF and DYMF are shown in Table 1, where it can be observed that all the results were closely similar to each other. The removal of the fat from foxtail millet did not significantly affect the protein content. Nonetheless our results corroborated the results reported for kodo millet [24]. Before defatting the fat content for foxtail millet white and yellow was 2.38% and 2.90%, respectively, but a significant decrease was observed for DWMF and DYMF (0.41% and 0.66%, respectively, Table 1). The fat content was relatively low when compared to pearl millet (7.6%) and quinoa (6.3%) [25]. It was observed that the fibre content was relatively low and carbohydrates content was significantly higher for all the samples (Table 1). The results from this work were within the range reported for other plant proteins studied [26]. Other authors found that the carbohydrates were mainly composed of sugars (sucrose and raffinose), fibers, pentosans, and starch [26].

### 3.2. Protein content of Foxtail Millet Protein Concentrates

The protein content of the white and yellow millet protein concentrates obtained have a protein content of 80.04 and 75.69% respectively. The millet protein concentrates were used for the investigation of *in vitro* trypsin digestibility, amino acid composition, protein solubility, thermal properties, molecular size, emulsifying, foaming, water and oil holding and bulk density properties.

### 3.3. Amino Acid Analysis

The essential amino acid composition of foxtail millet flour, millet protein concentrates and soy protein concentrate are shown in Table 2, along with the essential amino acid composition according to the 2007 FAO/WHO/UNU requirements. It is clear that the millet protein concentrates contains all the essential amino acids in good proportion as compared to the soy protein concentrate. It is also comparable with the FAO/WHO/UNU requirement of amino acids (2007). The results in Table 2 indicate that the amino acid composition of millet concentrate closely resembles that of the flour from which it was prepared. It was observed from (Table 2) that leucine and phenylalanine + tyrosine are in excess amounts in millet protein. Lysine is the first limiting amino acid in cereal as it was observed to be high in the present study and cysteine was low in both varieties (Table 2).

### 3.4. Sodium Dodecyl Sulfate Polyacrylamide Gel Electrophoresis (SDS-PAGE)

**Figure 1.**
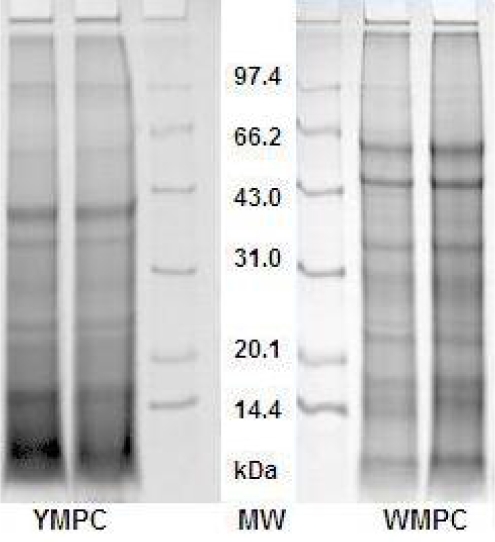
SDS-PAGE patterns of two varieties of foxtail millet protein concentrates. YMPC: Yellow millet protein concentrate; MW: Molecular weight marker; WMPC: White millet protein concentrate.

WMPC and YMPC revealed polypeptides of a wide range of molecular weights. WMPC and YMPC showed slight variation in the banding patterns. WMPC contain about six polypeptides with estimated molecular weight ranged from 14.4 to less than 66.2 kDa (Figure 1). However, five major polypeptides with estimated molecular weights around 14.4, 20.1, 31.0, 43.0 and 66.2 kDa were identified in the WMPC sample, while YMPC showed four intense polypeptides with estimated molecular weights around 14.4, 20.1, 31.0 and 43.0 kDa (subjectively judged, based on band width and intensity). The results are contrary to kodo millet and barnyard millet [27].

### 3.5. In Vitro Trypsin Digestibility

*In vitro* trypsin digestibility of WMPC and YMPC were determined and compared to that of SPC (Table 3). WMPC was more easily digested than YMPC. SPC, WMPC and YMPC had digestibility values with trypsin of 84, 81 and 78% respectively, showed a significant difference (*P* < 0.01) Table 3. Compared to SPC, WMPC and YMPC, showed a higher digestibility. It was mainly composed of albumin and globulin, which were soluble in solution and easily attacked by trypsin. The unfolding of the native protein structure during the cause of hydrolysis is yet another factor that likely facilitates digestibility [28].

### 3.6. Protein Solubility

**Figure 2.**
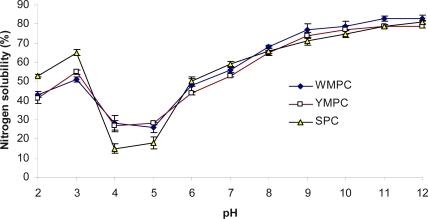
Effect of pH treatment on nitrogen solubility of foxtail millet protein concentrates and soy proteins concentrate. WMPC: White millet protein concentrate; YMPC: Yellow millet protein concentrate; SPC: Soy protein concentrate Value represent the mean ± standard deviation (SD) of n = 3 duplicate assays.

The pH-protein solubility profiles of WMPC, YMPC and SPC had very similar solubility profiles, exhibiting a U-shaped curve in which the WMPC had highest solubility values at alkaline pH. In acidic condition, all proteins had solubility (above 40%) at pH 2.0, but SPC has lower solubility than WMPC and YMPC at pH 4.0 and pH 5.0 (less than 20%). At pH 6.0 or above, all proteins dissolved between (50% and 80%), with WMPC having slight higher solubility than SPC and YMPC (Figure 2). These trends in solubilities corroborated with the data reported by Zahra *et al.* [29]. The maximum solubility was in alkaline conditions. Protein solubility at various pH values may serve as a useful indicator of how well protein concentrate will perform when they are incorporated into food systems. The solubility curve is typical to that of most seed proteins.

### 3.7. Foam Capacity and Foam Stability

Foamability is a function of the configuration of protein molecules. The formation of protein based foams involves the diffusion of soluble proteins toward the air-water interface and rapid conformational change and rearrangement at the interface; the Foam stability requires formation of a thick, cohesive, and viscoelastic film around each gas bubble [30]. To have foam stability, protein molecules should form continuous intermolecular polymers enveloping the air bubbles, since intermolecular cohesiveness and elasticity are important to produce stable foams. The Foam Capacity of WMPC was significantly higher (*P* < 0.01) than that of SPC. The foam capacity WMPC, SPC and YMPC, ranged from 137, 124 and 124 g/mL, respectively (Table 4). Similarly, foam obtained from YMPC was significantly less stable than that from SPC. The foam stability values ranged from 137 to 73, 124 to 61 and 124 to 46 g/mL WMPC, SPC and YMPC respectively, and the foam stability of millet protein concentrates were lower when compared to Singh *et al.* [31]. These foaming properties suggest that WMPC is a better foaming agent in protein food than SPC and YMPC.

### 3.8. Emulsifying Capacity

SPC have significantly lower emulsifying activity than WMPC and YMPC (Table 3). Emulsifying activity of WMPC and YMPC and SPC ranged from 74, 65 and 53%, respectively, but a significant difference (*P* < 0.01) were observed between WMPC, YMPC and SPC. Petruccelli *et al.* [32] reported that emulsifying capacity is closely associated with protein surface hydrophobicity. Furthermore, proteins are composed of charged amino acids, non charged polar amino acids and nonpolar amino acids, which make proteins possible emulsifiers [33]. The high emulsifying capacity of millet protein concentrates may be because of the surface tension of millet protein which contains high hydrophobic residues as obtained from Tang *et al.* [34], which can disperse the droplets of oil in aqueous continuous phase of the solution. Our data showed that WMPC, YMPC and SPC have relatively good emulsifying capacity properties and WMPC had highest emulsifying capacity. This could be due to the exposure of hydrophobic groups of denatured proteins.

### 3.9. Water/Oil Absorption

The water absorption capacity (WAC) of the WMPC, YMPC and SPC ranged from 7, 6 and 5 g/g, respectively (Table 3), but were significantly different (*P* < 0.01). SPC possessed the lowest water binding capacities (5 g/g), followed by YMPC (6 g/g) and WMPC had the highest (7 g/g). Chandi and Sogi reported much lower values of 3.87 g/g [13]. Interactions of water and oil with proteins are very important in the food systems because of their effects on the flavor and texture of foods. Intrinsic factors affecting water binding of food protein include amino acids composition, protein conformation and surface hydrophobicity/polarity [35]. In food applications, the water-holding capacity or water-uptake capacity of a protein is more important than hydration.

For oil absorption capacity WMPC and SPC have the highest 6 and 6 respectively, and YMPC had the lowest 5 g/g oil absorption capacities (Table 3) but were significantly different (*P* < 0.01). WMPC possessed oil absorption capacity similar to SPC (Table 3). Millet protein concentrates had lower oil absorbing capacity as compared to Singh *et al.* [31] Further more, high oil absorption is essential in the formulation of food systems like sausages, cake, batters, mayonnaise and salad dressings.

### 3.10. Bulk Density

SPC and YMPC had similar bulk density of 0.15 and 0.17 g/mL, respectively but was significantly different (*P* < 0.01) (Table 3). WMPC had the highest bulk density (0.22 g/mL) among the various protein concentrates. Millet protein concentrate had higher bulk density as compared to soy protein concentrate and rice bran protein concentrates [13]. Several authors have attributed solubility, hydrodynamic properties, hydrophobicity and microstructure of proteins plays an important role in the bulk density of any protein concentrate [36]. More over, bulk density is an important parameter that determines the packaging requirement of a product. Present results of SPC, YMPC and WMPC 0.15, 0.17 and 0.22 g/mL respectively, were in consistent with [37]. Bulk density signifies the behavior of a product in dry mixes. Also it varies with the fineness of the particles. High bulk density is disadvantageous for the formulation of weaning foods, where low density is required.

### 3.11. Differential Scanning Calorimetry (DSC)

DSC is a rapid, easy, and capable technique for supplying both thermodynamic (heat capacity, enthalpy, and entropy) and kinetic data (reaction rate and activation energy) on protein denaturation, and has been used extensively in various food systems. The information on protein thermal properties is useful for food-processing strategies and heat-processing design [38]. Data from DSC measurements for protein concentrates of YMPC, SPC and WMPC are given Table 4. According to the results the samples have varied denaturation temperatures of 88.98, 88.31 and 86.79 °C, respectively. The enthalpy differs among both varieties. Wang *et al*. [39] reported that the denaturation temperature and enthalpy changes were 83.4 °C and 0.96 J/g for rice bran protein isolate. The enthalpies of the various samples as stated above were, 0.01, 0.01 and 0.10 respectively. In this study, the various protein concentrate where less denatured than similar product [39].

## 4. Conclusions

Defatted foxtail millet protein concentrates from both varieties (white and yellow) exhibited substantial amounts of protein content. Furthermore, the results of the protein characterization show that millet protein concentrate is a potential functional food ingredient. SPC was more digestible than WMPC and YMPC. The essential amino acid pattern of foxtail millet protein concentrates suggests their possible use as a supplementary protein source to most cereals because this protein is rich in lysine, which is the first limiting amino acid in most cereals. The two concentrates varied in their denaturation temperatures and revealed a wide range of molecular weights polypeptides. The three samples showed lower solubility at pH 4.0 and 5.0. WMPC exhibited the highest solubility. WMPC and YMPC have higher emulsifying capacity, foam capacity and stability than SPC. Millet protein concentrates thus have excellent applications for future product development by virtue of their functional properties.

## Figures and Tables

**Figure 3.**
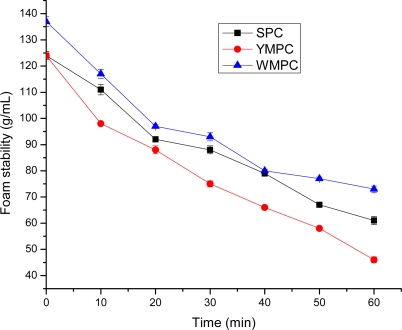
Foam stability of foxtail millet protein concentrates and soy proteins concentrate. WMPC: White millet protein concentrate; YMPC: Yellow millet Protein concentrate; SPC: Soy protein concentrate Value represent the mean ± standard deviation (SD) of n = 3 duplicate assays.

**Table 1.** Proximate chemical composition of two varieties of foxtail millet flour and defatted foxtail millet flour (white and yellow) (g/100 g, dry basis).

**Sample**	**Protein**	**Moisture**	**Fat**	**Ash**	**Crude fiber**	**Carbohydrate**
WMF	11.50 ± 1.08	10.45 ± 0.14	2.38 ± 0.03	0.47 ± 0.03	1.88 ± 0.01	73.33 ± 0.03
YMF	11.41 ± 0.15	10.22 ± 0.13	2.90 ± 0.35	0.68 ± 0.04	1.92 ± 0.02	73.00 ± 0.14
DWMF	11.92 ± 0.30	12.23 ± 0.04	0.41 ± 0.15	0.44 ± 0.04	1.95 ± 0.02	72.92 ± 0.01
DYMF	11.39 ± 0.38	12.09 ± 0.10	0.66 ± 0.17	0.91 ± 0.03	2.02 ± 0.10	72.91 ± 0.08

Values are mean ± standard deviation of four determinations; WMF: White millet flour; YMF: Yellow millet flour; DWMF: Defatted white millet flour; DYMF: Defatted yellow millet flour.

**Table 2.** Comparative amino acid profiles of two varieties of defatted foxtail millet flour (DWMF & DYMF), millet protein concentrates (WMPC & YMPC) and commercial soy protein concentrate (SPC) g/100 g of protein.

**Essential Amino Acids**	**DWMF**	**DYMF**	**WMPC**	**YMPC**	**SPC**	**FAO/WHO/UNU^a^**	
						**Child**	**Adult**
Isoleucine (Ile)	4.58	4.59	3.82	3.91	5.18	3.0	3.0
Leucine (Leu)	13.14	13.60	8.58	8.71	8.13	6.0	5.9
Lysine (lys)	3.43	3.85	5.17	6.07	6.74	4.8	4.5
Methionine (Met)	2.72	3.06	2.66	2.42	1.99		
Met + Cys	3.06	3.50	3.79	3.15	2.49	2.3^b^	1.6^b^
Phenylalanine (Phe)	7.73	6.27	5.21	5.34	5.54		
Phe + Tyr	10.68	8.71	9.33	9.00	8.79	4.1^c^	3.8^c^
Threonine (Thr)	2.76	3.68	4.28	4.55	3.57	2.5	2.3
Valine (Val)	5.58	5.81	5.74	5.79	5.57	2.9	3.9
Histidine (His)	2.06	2.11	3.01	2.80	2.54	1.6	1.5
Tryptophan (Trp)	1.14	1.39	1.51	1.53	0.02	0.66	0.6
**Nonessential Amino Acids**							
Alanine (Ala)	10.89	9.30	6.15	6.35	4.29		
Arginine (Arg)	4.91	4.78	8.63	7.86	7.83		
Aspartic acid (Asp)^d^	6.51	7.71	8.49	8.97	11.27		
Cysteine (Cys)^e^	0.34	0.45	1.23	0.74	0.50		
Glutamic Acid (Glu)^f^	23.77	22.00	14.97	14.08	21.04		
Glycine (Gly)	2.22	2.91	5.06	5.21	4.23		
Serine (Ser)	5.17	4.56	5.14	5.19	2.54		
Tyrosine (Tyr)	2.94	2.44	4.11	3.66	3.35		
Proline (Pro)	5.10	5.54	5.38	6.49	3.58		

a FAO/WHO/UNU energy and protein requirements (2007);

b Requirements for methionine + cysteine;

c Requirements for phenylalanine + tyrosine;

d Aspartic acid + asparagines;

e Cysteine + cystine;

f Glutamic acid + glutamine.

**Table 3.** *In vitro* digestibility, emulsifying capacity, foam capacity, water holding capacity, oil holding capacity and bulk density.

	SPC*	YMPC**	WMPC***
*In vitro* digestibility (%)	84 ± 1.15^b^	78 ± 0.58^a^	81 ± 1.53^ab^
Emulsifying capacity (%)	53 ± 1.2^a^	65 ± 1.50^b^	74 ± 1.00^c^
Foam capacity (g/mL)	124 ± 1.53^a^	124 ± 1.00^a^	137 ± 2.00^b^
Water holding capacity (g/g)	5 ± 0.07^a^	6 ± 0.06^b^	7 ± 0.15^c^
Oil holding capacity (g/g)	6 ± 0.22^ab^	5 ± 0.09^a^	6 ± 0.61^b^
Bulk density (g/mL)	0.15 ± 0.03^a^	0.17 ± 0.01^ab^	0.22 ± 0.02^b^

Values are means ± standard deviation of three determinations; Rows with different letters indicate statistical differences (*P* < 0.01);

* Soy protein concentrate;

** Yellow millet protein concentrate;

*** White millet protein concentrate.

**Table 4.** Thermal properties of two varieties of defatted foxtail millet protein concentrates and commercial soy protein concentrate.

	**DSC Measurea(**°**C)**				

**Protein source**^b^	**To**	**Tp**	**Te**	**ΔH J/g**	**Ar. mJ**
**WMPC**	84.06	86.79	88.29	0.10	0.15
**YMPC**	87.55	88.98	88.98	0.01	0.03
**SPC**	86.25	88.31	88.84	0.01	0.03

a To: Start Temperature Peak, Tp: Peak Temperature, Te: End Temperature,ΔH: Delta H, Ar mJ: area;

b WMPC: White millet protein concentrate; YMPC: Yellow millet protein concentrate; SPC: soy protein concentrate.
